# What COVID-19 means for non-neurotypical children and their families

**DOI:** 10.1038/s41390-020-0913-7

**Published:** 2020-04-21

**Authors:** Michele Kong

**Affiliations:** The University of Alabama at Birmingham, Birmingham, AL USA

Coronavirus disease 2019 (COVID-19) is a novel respiratory infection caused by the virus severe acute respiratory syndrome coronavirus 2 (SARS-CoV-2), which has over a short period of time become a major global health threat (https://www.cdc.gov/coronavirus/2019-ncov/index.html). As of 21 April 2020, there is over 2,498,000 confirmed cases worldwide, with more than 171,000 death globally (https://coronavirus.jhu.edu/map.html). The world struggles to meet the demands imposed by the virus, not only on the health care system but also the economy as well. While research is fervently underway to develop a vaccine and/or treatment against the virus, social distancing becomes our main course of defense (https://www.cdc.gov/coronavirus/2019-ncov/downloads/community-mitigation-strategy.pdf). Schools, museums, zoos, libraries, restaurants, and even national parks are closed, and individuals are asked to stay home, except for essential activities.

COVID-19 has and will continue to change our way of life. Parents adapt to working remotely from home and children learn to use online learning platforms. Outings and outside activities are no longer the routine, and only limited to within the family unit. These sudden, unexpected, and drastic lifestyle changes will be hard for any individual, especially for non-neurotypical children, such as those with autism. During this time, it is critical for us to have an awareness of the additional challenges these children and their family will face. This is a vulnerable patient group, who in addition to their health problems has additional barriers related to their primary developmental, social, communication, and sensory challenges, which will be heightened during the COVID-19 pandemic.

Children with autism often have difficulties with communication and may have a harder time understanding the rapidly changing situation.^1^ Additionally, they may struggle with expressing their feelings. Limitations in understanding, coupled with expressive delay and impairments in social reciprocity, may lead to maladaptive behaviors such as self-aggressions. Furthermore, these children favor predictable routines and struggle with schedule changes. Because of their insistent on sameness, and preference for certain activities or places, the inability to do their preferred activity may compound the frustration of being home bound.

By virtue of their chronic health issues, functional limitations, or technology dependence, children with medical conditions often have a large team of providers. They have unique needs that depend on services from physicians, occupational, speech, and physical therapist, to special education teachers and specialist. With closure of schools, and limitation of clinics to only essential services (an acute illness for instance versus routine rehabilitation session), these children will lose a major component of their support system. They become at risk for no progress, or worst, regression of skills or milestones. They often also have sensory processing difficulties that can range from discomfort to bright lights and loud noises to disturbed sensitivity to proprioceptive and vestibular inputs that leads to social deficit and isolation.^2^ Now, these children and their family, who are already isolated from public life, are being asked to distance themselves even further, potentially heightening and compounding their isolation.

During this time, parents and caregivers must adapt to new roles, reorganize their lives, and learn to cope with the increased care demands. It is important to establish a new home routine. Ensure that the wake and sleep time is consistent. Schedule specific times for schoolwork, meals, play time, and exercise. Keeping a consistent schedule, albeit new and different will become the norm, alleviate anxiety, and help with the adjustment period. Visual daily calendars can be helpful to delineate passing of time, and to aid the child in anticipating the next agenda for the day.

In order to facilitate understanding, it is crucial that caregivers keep the language simple, clear, and concrete. For example, a child with autism is more likely to understand “I am sick” rather than “I am under the weather” or “I caught the virus”. Visual cues can be used to teach certain behaviors such as handwashing. Story boards that describe situations or an encounter in a precise and sequential way using simple and literal language can also be used to facilitate communication.

As medical providers and as a community, it is important for us to have an appreciation of the additional challenges that non-neurotypical children and their families face. Even with clinic cancelations and absence of routine visits, it is crucial that we remain connected to them, and for them to know that they are supported. We need to innovate and utilize telemedicine to meet as much of their needs as we can. For parents, they need to know that they are not alone in this fight. The human spirit is resilient, and if we are ready to forgive ourselves for imperfect days, and soldier on, we will arrive at the end whole.
